# Correction: Diversity and community distribution of soil bacterial in the Yellow River irrigation area of Ningxia, China

**DOI:** 10.1371/journal.pone.0328921

**Published:** 2025-07-22

**Authors:** Xia Wu, Jinjun Cai, Zhangjun Wang, Weiqian Li, Gang Chen, Yangyang Bai

The word “Bacteria” is misspelled in the article title. The correct title is: Diversity and community distribution of soil bacteria in the Yellow River irrigation area of Ningxia, China. The correct citation is: Wu X, Cai J, Wang Z, Li W, Chen G, Bai Y (2024) Diversity and community distribution of soil bacteria in the Yellow River irrigation area of Ningxia, China. PLoS ONE 19(9): e0311087. https://doi.org/10.1371/journal.pone.0311087
