# Supplementary material for: Diversity and community distribution of soil bacterial in the Yellow River irrigation area of Ningxia, China
Source: PLoS One. 2024 Sep 30;19(9):e0311087. doi: 10.1371/journal.pone.0311087 (PMC11441701; doi:10.1371/journal.pone.0311087)
Supplement: Attachment — Submitted filename: Response to Reviewers.docx [file pone.0311087.s007.docx]

**Dear Editors:**

Thank you for your decision and constructive comments on our manuscript. We have carefully considered the suggestion of Reviewer and make some changes. We have tried our best to improve and made some changes in the manuscript. Revision notes are given as follows:

1. Revise the manuscript according to the PLOS ONE's style requirements.
2. The data used for manuscript collection is obtained through on-site investigation and sampling by the project team in the research area, soil physical and chemical properties, and testing, without the need for a permit.
3. The R code for the drawing will be uploaded after acceptance.
4. The Ningxia Hui Autonomous Region Agricultural High Quality Development and Ecological Protection Technology Innovation Demonstration Project (NGSB-2021-11-01) and the Ningxia Hui Autonomous Region Key R&D Project (2023BEG02042) jointly provide funding support for research work, including soil sampling investigation, soil sample testing, data collection and analysis, decision to publish, preparation of the manuscript. The Guangzhou Genedenovo Biotechnology Co., Ltd assists in soil bacterial testing and analysis.
5. All data used in the submission has been listed with file names in the revised manuscript and has been submitted.
6. Delete Figure 1 from the manuscript, which includes a map. Deleting Figure 1 does not affect the description of the sampling area in the manuscript, as Table 1 already lists the specific information of the sampling area.

We look forward to your information about our revised manuscript. Once again,thank you very much for your comments and suggestions.

Best regards,

Yours sincerely ,

Xia Wu

Corresponding Author:

Jinjun Cai

No. 590, Huanghe East Road, Yinchuan, Ningxia, 750002, China.

Email address: [nxyccai@163.com](mailto:nxyccai@163.com)
